# Wireless Intelligent Patch for Closed‐loop In Situ Wound Management

**DOI:** 10.1002/advs.202400451

**Published:** 2024-06-03

**Authors:** Zijian Liu, Hao Song, Guanming Lin, Weicong Zhong, Yang Zhang, Anqi Yang, Yuxin Liu, Junhan Duan, Yajing Zhou, Kangjian Jiao, Donghai Ding, Yanwen Feng, Jun Yue, Wenjing Zhao, Xudong Lin

**Affiliations:** ^1^ Guangdong Provincial Key Laboratory of Sensor Technology and Biomedical Instrument School of Biomedical Engineering Shenzhen Campus of Sun Yat‐Sen University Shenzhen 518000 China; ^2^ Shenzhen Key Laboratory for Systems Medicine in Inflammatory Diseases School of Medicine Shenzhen Campus of Sun Yat‐Sen University Shenzhen 518000 China

**Keywords:** bioelectronics, DNA hydrogel, electrical stimulation, OECT, wound management

## Abstract

Wound infections pose a major healthcare issue, affecting the well‐being of millions of patients worldwide. Effective intervention and on‐site detection are important in wound management. However, current approaches are hindered by time‐consuming analysis and a lack of technology for real‐time monitoring and prompt therapy delivery. In this study, a smart wound patch system (SWPS) designed for wireless closed‐loop and in‐situ wound management is presented. The SWPS integrates a microfluidic structure, an organic electrochemical transistor (OECT) based sensor, an electrical stimulation module, and a miniaturized flexible printed circuit board (FPCB). The OECT incorporates a bacteria‐responsive DNA hydrogel‐coated gate for continuous monitoring of bacterial virulence at wound sites. Real‐time detection of OECT readings and on‐demand delivery of electrical cues to accelerate wound healing is facilitated by a mobile phone application linked with an FPCB containing low‐power electronics equipped with parallel sensing and stimulation circuitry. In this proof‐of‐concept study, the functionality of the SWPS is validated and its application both in vitro and in vivo is demonstrated. This proposed system expands the arsenal of tools available for effective wound management and enables personalized treatment.

## Graphical abstract

In this work, Liu and co‐workers develop a smart wound patch system (SWPS) consisting of microfluidic structures, an organic electrochemical transistor‐based sensor, an electrical stimulation module, and a miniaturized flexible printed circuit board, to enable wireless, closed‐loop, and in situ wound management. The functionalities and applications of the SWPS are successfully demonstrated both in vitro and in vivo.

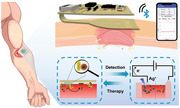

## 1 Introduction

The skin, the largest organ in the human body, plays a crucial role in defending against injuries and facilitating wound healing.^[^ ^1^ ^]^ However, various factors, including severe trauma, extensive burns, and chronic diseases like diabetes can compromise the skin's defensive and regenerative abilities, leading to the emergence of chronic wounds.^[^ ^2^ ^]^ Healing these wounds is a complicated process often marked by prolonged and uncontrolled inflammation, pathogenic bacterial infections, and cellular aging, posing a significant global medical challenge and healthcare burden.^[^ ^2^, ^3^ ^]^ Of particular concern is the presence of microbial infections at chronic wound sites, which can severely prolong healing time and increase the risk of complications such as sepsis, multiple organ dysfunction syndrome, and mortality.^[^ ^4^ ^]^ Therefore, timely detection of bacterial infection and effective management of chronic wounds are very important to promote healing and improve patient outcomes.^[^ ^5^ ^]^

Current therapies for chronic wounds typically employ traditional wound dressings and systemic antibiotic treatments to expedite wound healing.^[^ ^6^ ^]^ Traditional dressings, such as bandages, gauzes, and cotton, while effective in absorbing fluids and exudates from wound sites, have limitations in providing an anti‐bacterial environment conducive to infection prevention and skin regeneration.^[^ ^7^ ^]^ Although recent proposals have introduced dressings made from biological materials such as collagen, chitosan, and silk to enhance skin repair, the absence of effective infection detection in these strategies, which rely on time‐consuming culture‐based assays, leads to delays in administering appropriate treatment.^[^ ^8^ ^]^ Moreover, the lack of continuous infection monitoring often leads to the inappropriate use of antibiotics, contributing to the development of antimicrobial resistance, and thereby significantly increasing morbidity and mortality rates.^[^ ^9^ ^]^

To overcome these issues, tremendous efforts have been dedicated to developing patch systems for in situ sensing and treatment of chronic wounds.^[^ ^4^, ^10^ ^]^ For example, various patches incorporating biochemical sensors have been proposed to enable sensitive, real‐time monitoring of changes in potential hydrogen (pH), glucose levels, and temperature at wound sites.^[^ ^10^, ^11^ ^]^ Moreover, advancements in bioelectronics have led to the creation of wearable integrated systems capable of simultaneous monitoring and treatment of chronic wounds.^[^ ^10^, ^12^ ^]^ Nevertheless, many of these systems primarily focus on physiological profiling, such as thermal and impedance detection, which do not directly assess bacterial virulence. While optical and electrochemical strategies have been suggested for detecting specific markers of pathogenic bacteria, the complexity of readout modules hinders their integration into wearable patch systems.^[^ ^13^ ^]^ Recently, it was demonstrated that a sensing technology based on bioelectronics and bacteria‐responsive DNA hydrogel can directly detect *Staphylococcus aureus* (*S. aureus*) infection in vivo.^[^ ^8a^ ^]^ However, achieving wound treatment functionality with this system remains unexplored. Therefore, the development of an intelligent wound patch capable of real‐time, continuous, and on‐site monitoring of bacterial virulence, coupled with closed‐loop management of chronic wounds, holds significant promise and warrants further exploration.

Here, we present the design of a smart wound patch system (SWPS) for wireless, closed‐loop, in‐situ management of chronic wounds. The core of the proposed system is an integrated device consisting of a microfluidic structure, an organic electrochemical transistor (OECT)‐based sensor, Ag electrodes, and a miniaturized flexible printed circuit board (FPCB). To enable continuous monitoring of bacterial virulence at wound sites, the SWPS utilizes an OECT with a DNA hydrogel‐coated gate integrated into the flexible patch system. Upon exposure to deoxyribonuclease (DNase), an extracellular enzyme secreted by bacterial pathogens including *S. aureus*, the channel current of the OECT can be modulated by the gate voltage due to DNAgel degradation. Real‐time monitoring of OECT readouts and on‐demand delivery of electrical stimulation to accelerate wound healing are achieved through the FPCB, which incorporates low‐power electronics with parallel stimulation and sensing circuit modalities. In this proof‐of‐concept study, we validate the functionality and demonstrate the applications of the SWPS for wound infection monitoring and healing both in vitro and in vivo. We envision that our SWPS device will expand the toolkit available for wound management and further facilitate personalized optimization of treatment modalities to improve patient mobility.

## 2 Results

### 2.1 System Design of SWPS

**Figure 1:**
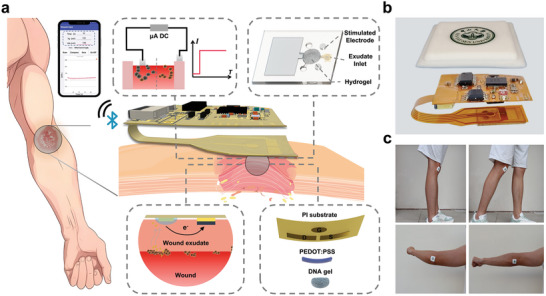
Design of SWPS. a) Schematic of the smart wound patch system (SWPS) for wireless, continuous, and closed‐loop wound management. b) Images showing an exploded view of SWPS, including silicone encapsulation layer, FPCB layer, OECT layer, and microfluidic layer. The device is ultimately encapsulated in silicone. c) Photographs showing SWPS firmly adhered to elbow and knee during movements.

To enable wireless, continuous monitoring, and closed‐loop treatment of chronic wounds, we developed a smart wound patch system (SWPS) (**Figure** **1**a). It integrated multiple layers of functional structures, including microfluidic channels, an OECT‐based sensor, Ag electrodes, and a miniaturized FPCB, to create a fully integrated smart system (Figure 1a,b). As the core component of the SWPS, an OECT with a DNA hydrogel (DNAgel) coated gate was utilized to continuously monitor the bacterial virulence at wound sites. DNAgel could be degraded by exposure to deoxyribonuclease (DNase), an extracellular enzyme primarily secreted by bacterial pathogens such as *S. aureus*, leading to a fluctuation in the channel current (*I*_ds_) of the OECT. Based on the readouts from the OECT sensor, on‐demand electrical stimulation could be directly delivered to the wound via the Ag electrodes in the SWPS. Moreover, due to the thin profile of the FPCB (approximately 100 µm) and the soft‐encapsulation overlayer (silicone elastomer), the SWPS device exhibited stretchability, enabling it to be applied to wound management on various body locations, including joint areas such as elbow, knees, and wrists (Figure 1c; Figure S1, Supporting Information). With secure adhesion, our device could be affixed to any wound site, allowing patients or animals freedom of movement.

### 2.2 Circuit Design of FPCB

**Figure 2:**
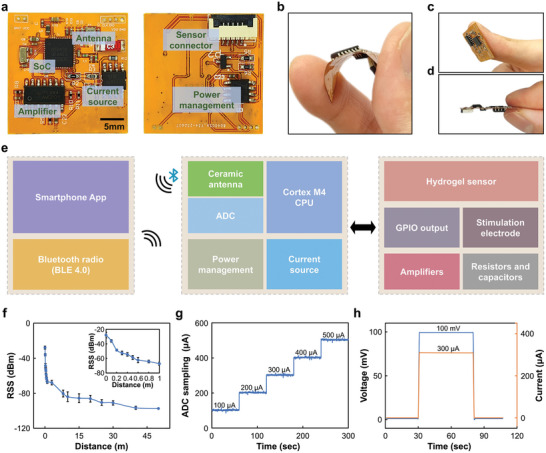
Design of FPCB module. a) Photographs of top and bottom views of FPCB, showing SoC, antenna, amplifier, current source, power management, and sensor connector. b–d) Photographs showing flexibility (b), miniaturized size (c), and thin board layout (d) of FPCB. e) Block diagram showing key components of SWPS, including Bluetooth low‐power electronics, hydrogel sensor, and electrical stimulation (ES) electrodes. f) received signal strength (RSS) analysis of Bluetooth signal in SWPS, showing reliable wireless communication within a distance of 50 m. g) Representative I/T curve showing a high analog‐to‐digital converter (ADC) sampling ability for precise differentiation of currents at microampere scale. h) Representative V/T and I/T curves showing precise voltages and currents delivered by signal generation module in FPCB for further ES.

**Figure** **2**a illustrates the overall view of the FPCB in the SWPS, which was based on a 12.5‐µm‐thick middle polyimide support layer with patterned traces of copper lines on the top and bottom surfaces, each encapsulated with an insulating layer of polyimide (27.5 µm). While electronic subcomponents such as the system‐on‐a‐chip (SoC), antenna, amplifier, and filter were rigid, the final miniaturized scale and flexibility of the FPCB allowed for comfortable attachment to the wound area (Figure 2b–d). The main electronic subsystems of the FPCB circuit comprised 1) a constant voltage/current excitation circuit and a current‐voltage conversion (I‐V converter) acquisition circuit; 2) a microcontroller (nRF52832, Nordic Semiconductor) for sampling the voltage signal from the OECT sensor and wirelessly communicating the results via Bluetooth low‐energy protocols (BLE 4.0); 3) a ceramic patch antenna with optimized impedance matching design; and 4) a power management module with a low dropout regulator (LDO) offering low noise and fast response, and a negative charge pump supplying a stable −3.3 V to the peripheral circuit (Figure 2e; Figure S2, Supporting Information). In comparison to near‐field communication (NFC) or radio‐frequency (RF)‐powered strategies in many current bioelectronics, the BLE design in SWPS provided ideal stability of working voltages, a secure data stream, and long communication distance. The Bluetooth signal in the device maintained a reliable received signal strength (RSS) value within a communication distance of 50 m under barrier‐free conditions (Figure 2f). Coupled with amplifier and analog‐to‐digital converter (ADC) modules in the FPCB, our device could record a current as low as 100 µA with a 10 µA sampling accuracy (Figure 2g). Importantly, our FPCB circuit ensured highly stable voltages to the source, drain, and gate of the OECT for wound monitoring and generated reliable on‐demand current excitation via a current source to the electrodes for wound healing (Figure 2h). All commands could be adjusted wirelessly via phone during further operation.

### 2.3 Sensor Design and Characterization

**Figure 3:**
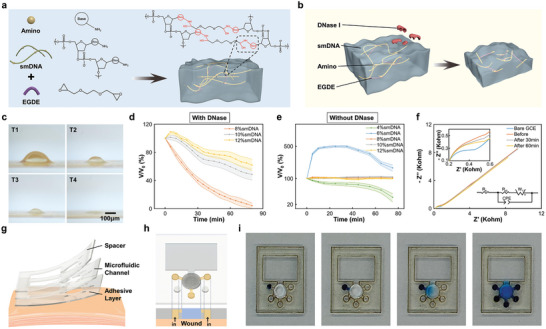
Characterization of DNA Hydrogel and microfluidic layers. a) Schematic showing synthesis of DNA hydrogel (DNAgel). b) Schematic showing DNase‐induced DNAgel ablation. c) Images showing changes of DNAgel shapes exposed to 0.1 U mL^−1^ DNase I. Imaging interval was 20 min. d,e) Relative volume changes of DNAgels (with different smDNA cross‐linking ratios from 4 to 12 wt.%) with (d) and without (e) DNase I (0.1 U mL^−1^) exposure. f) Electrochemical impedance spectroscopy (EIS) analysis of electrodes (with or without DNAgel coating) before and after DNase I exposure. GCE indicated bare glassy carbon electrode (blue); DNAgel‐coated GCE was analyzed before (orange) after 30 min (gray) and 60 min (yellow) exposure of DNase I. Frequency range spanned 0.1–100 kHz with Direct Current (DC) potential of 0.2 V and alternating current (AC) amplitude of 5 mV. g) Schematic of microfluidic structure was constructed from three thin layers of flexible patterned polyethylene terephthalate (PET). h,i) Schematic (h) and images (i) showing a collection of wound exudate (blue dye) and its interaction with DNA hydrogel.

To incorporate the bacteria‐responsive components into the device, we applied a DNAgel synthesized through a physicochemical cross‐linking method, as shown in **Figure** **3**a. Our synthesis strategy was fast (5 h), stable, and highly reproducible compared to previous works, including solely physical or chemical methods.^[^ ^14^ ^]^ It was well established that the DNAgel is selectively degraded by the extracellular DNase enzyme, commonly secreted by opportunistic pathogens such as *S. aureus* at the wound site^[^ ^8^, ^15^ ^]^ (Figure 3b).

We confirmed the DNase‐induced degradation of the DNAgel. As shown in Figure 3c,d, incubation with DNase I solution (0.1 U mL^−1^) for 1 h led to complete degradation of the DNAgel droplet with specific smDNA cross‐linking ratios (8 wt.%). Different proportions of smDNA induced varied pore structures, which could affect material interchanging rates of the DNAgel.^[^ ^16^ ^]^ And such DNAgel could be easily integrated into the bioelectronic interfaces, such as the OECT‐sensing module in the SWPS owing to its in‐situ gelation ability and high stability in an aqueous environment (Figure 3e; Figure S3, Supporting Information). Furthermore, we validated the electrical responses of the DNAgel to the DNase I enzyme via electrochemical impedance spectroscopy analysis (Figure 3f). The results showed that 0.5‐h incubation with a DNase I solution (0.1 U mL^−1^) could induce a 168 pF capacitance change of a glassy carbon electrode (GCE), which was uniformly coated with a 1.5 mm high cylindrical layer of DNAgel when applied the Randles equivalent circuit model. Such capacitance fluctuation of the DNAgel on the electrode surface could be further adapted to the OECT design for monitoring the bacterial infection in the chronic wound.

To establish an interface for infection signal transduction from the wound site, a flexible microfluidic structure comprising an OECT chamber, DNAgel chamber, and small chambers for wound exudate collection and electrical stimulation was designed in the SWPS device (Figure 3g,h). As shown in Movie S1 (Supporting Information), wound exudate would be effortlessly collected from the wound site using the capillary effect. In in vitro testing, the wound exudate could be easily collected and loaded into the DNAgel chamber to induce degradation of the DNAgel upon infection of the wound (Figure 3i).

**Figure 4:**
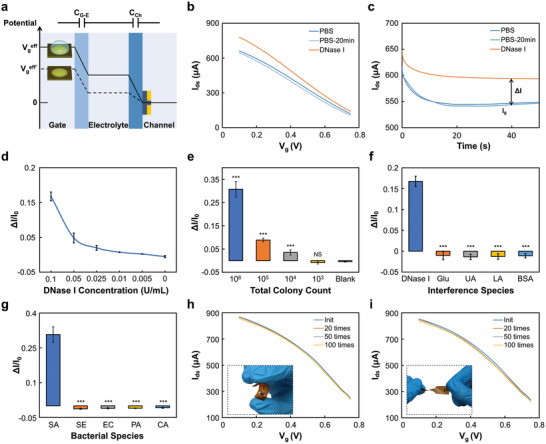
In vitro validation of Hydrogel Gated OECT for wound sensing. a) Schematic illustrating the working principle of hydrogel‐gated OECT. b,c) Electrical characterization of DNA hydrogel gated OECT, including representative transfer curves *I*_ds_–*V*_g_ (b) and time‐dependent *I*_ds_ curves (c) for exposure to PBS (initial and post‐20‐min), and DNase I at 0.1 U mL^−1^. Transfer characteristics with *V*_g_ range from 0.1 to 0.75 V and *V*_ds_ are set at 0.1 V (b). Time‐dependent *I*_ds_ responses are captured with both *V*_ds_ and *V*_g_ set at 0.1 V (c). d) Normalized transient deviation of *I*_ds_ from its baseline was evaluated with 20‐min interval. e) Quantification of OECT readouts (*I*_ds_ deviation) for *S. aureus* (*S. aureus*) suspensions with varying concentrations in PBS. f) Quantification of OECT readouts for various chemicals including DNase I (0.1 U mL^−1^), glucose (Glu, 5 mm), uric acid (UA, 300 µm), lactic acid (LA, 10 µm) and bovine serum albumin (BSA, 15 mm). g) Quantification of OECT readouts for various bacteria including *S. aureus*, *Staphylococcus epidermidis* (*S. epidermidis*), *Escherichia coli* (*E. coli*), *Pseudomonas aeruginosa* (*P. aeruginosa*), and *Cutibacterium acnes* (*C. acnes*). The concentrations of tested bacteria were 106 CFU mL^−1^. h,i) Transfer characteristics of OECT structure with repeated bending (h), and twisting (i). Two‐tailed paired student *t*‐test, *n* = 5, collected from five devices; NS, not significant, ^***^ *p* < 0.001; error bars indicated standard deviation.

Subsequently, the OECT‐sensing structure was integrated with a flexible microfluidic device to form the final micro‐sensor for in‐situ wound monitoring (Figure S4, Supporting Information). The OECT‐sensing structure was fabricated through selective sputtering and deposition on a flexible polyimide substrate, consisting of a gold GATE electrode coated with DNAgel and a poly(3,4‐ethylenedi‐oxythiophene):poly(styrene‐sulfonate) (PEDOT:PSS) thin semiconducting film bridging the gold source and drain electrodes (Figure S5, Supporting Information). The working mechanism of the DNAgel gating effect on the OECT was explicitly illustrated in **Figure** **4**a. When a gate voltage *V*_g_ was applied, it was mainly distributed across the two electric double layers at the gate/electrolyte and channel/electrolyte interfaces, which could be considered as two capacitors: *C*_g‐e_ and *C*_Ch_. In the absence of DNase, the potential drop at the channel/electrolyte interface *V*_Ch_ was *V*_g_/(1+*C*_Ch_/*C*_g‐e_). Upon exposure to DNase, *V*_Ch_ decreases due to DNAgel degradation‐induced changes in *C*_g‐e_, leading to an increase in *I*_ds_.^[^ ^17^ ^]^ Using *I*_ds_ as one of the output parameters, the DNase‐sensing performance was evaluated (Figure S6, Supporting Information). The *I*_ds_ – *V*_g_ transfer curves showed that *I*_ds_ increased with the addition of DNase I enzyme (Figure 4b). Particularly, a notable increase in *I*_ds_ was observed when a low *V*_g_ (0.1 V) was applied (Figure 4c). We also examined the deviation of *I*_ds_ (△*I*_ds_) as a function of the coincubation of DNase I enzyme concentrations. The results showed that △*I*_ds_ were adjusted by varying the DNase I concentration (Figure 4d). Additionally, the electrical readouts (△*I*_ds_/*I*_0_) of the OECT were confirmed to increase with the *S. aureus* concentrations in vitro, showing the sensitivity of the OECT in infection monitoring (Figure 4e). To further validate the detection accuracy of our OECT system, we compared our results with those of a commercially available enzyme‐linked immunosorbent assay (ELISA) kit for DNase I. As shown in Figure S7 (Supporting Information), the results from both strategies exhibited a high positive correlation, with Pearson correlation coefficients of 0.9897 for the DNase I samples and 0.9928 for the SA suspension samples. This indicates that the detection accuracy of the OECT‐based strategy was comparable to that of the ELISA‐based method. Moreover, to verify the sensing selectivity of the OECT, a series of physiologically relevant electroactive molecules were employed to evaluate the specificity of our device. As shown in Figure 4f, our system exhibited high selectivity for DNase (0.1 U mL^−1^) detection against glucose (Glu, 5 mm), uric acid (UA, 300 µm), lactic acid (LA, 10 µm), and bovine serum albumin (BSA, 15 mm). Interestingly, a comparative analysis of the OECT sensor's response to *S. aureus*, *Staphylococcus epidermidis* (*S. epidermis*), *Escherichia coli* (*E. coli*), *Pseudomonas aeruginosa* (*P. aeruginosa*), and *C. acnes* (*C. acnes*) indicated high sensing selectivity for SA in the SWPS (Figure 4g). This highly selective sensing capability was advantageous for infection monitoring in vivo, particularly in environments with high interference. While the SWPS device was specifically designed to meet the requirements of complex wound applications, such as wounds on joints, we conducted various cycling tests to validate the mechanical and electrical stability of the OECT structure (Figure 4h,i; Figure S8, Supporting Information). In addition, long‐term, in‐situ monitoring of wounds was anticipated using our system, underscoring the importance of the reproducibility and stability of the OECT. In this proof‐of‐concept study, no significant changes were observed in the OECT readouts during the one‐week stability test (Figure S9, Supporting Information). Taken together, these results showed that the DNAgel‐gated OECT structure could effectively serve as a wound infection sensor with high sensitivity, selectivity, and stability.

### 2.4 Electrical Stimulators in SWPS

Electrical stimulation has been shown to reduce bacterial colonization, improve tissue perfusion, boost immune function, and promote normal wound healing in vivo.^[^ ^18^ ^]^ Therefore, to accelerate wound healing during infection, we integrated an electrical stimulation module into the SWPS. To ensure efficient delivery of electrical charges to the wound site, two Ag electrodes with low contact impedance were integrated into the microfluidic device and connected to the FPCB (Figure S4, Supporting Information). Cyclic voltammetry (CV) analysis of an Ag electrode immersed in DPBS (pH 7.4) at room temperature showed no additional redox response except for the normal oxidation observed across voltages ranging from 0 to 0.8 V (Figure S10a, Supporting Information). In in vitro tests, the delivery of a 300 µA (DC) electrical stimulus from our device significantly inhibited the growth of *S. aureus* on LB agar plates (Figure S11, Supporting Information). The adjustable charge injections of our device remained well maintained even after three weeks of repetitive stimulation (Figure S10b, Supporting Information). These results indicated that our SWPS platform, combining wound monitoring and infection inhibition, could be further utilized in the closed‐loop management of wounds.

### 2.5 Closed‐Loop Wound Management in Rodent Animals

**Figure 5:**
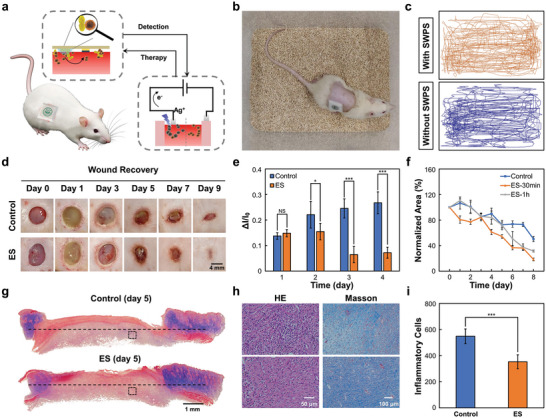
In vivo wound management with SWPS. a) Schematic of SWPS for wound management. b) Photograph of freely moving rat with SWPS. c) Moving trajectories of rats with and without SWPS in 1 h. d) Photographs of wound closure showing the progression of wound regeneration without (Control) and with electrical stimulation (ES) treatment in nine days. e) A quantitative study of OECT readouts over four days after *S. aureus* colonization in wound sites. f) Comparison of normalized wound area changes between the control group and ES group with 30‐min or 1‐h electrical stimulation. g) Representative images showing Masson's trichrome‐stained skin tissue samples from rats. h) Sections of granulation tissue (dashed boxes in g) stained with HE and Masson's trichrome showing distribution of inflammatory cells and collagen fibers. i) Quantification of inflammatory cells in granulation tissue (per 100 000 µm2) in control and ES groups. Paired samples *t*‐tests, *n* = 5; NS, not significant, ^*^ *p* < 0.05, ^***^ *p* < 0.001; error bars indicated standard deviation.

To validate the utility of the SWPS device for infection monitoring and wound healing, in vivo evaluation in rodents was essential. We employed our device in an acute wound rat model for in‐situ DNase enzyme monitoring and electrical stimulation therapy (**Figure** **5**a). Briefly, full‐thickness bilateral excisional wounds (≈8 mm in diameter) were created through the panniculus carnosus on the dorsum on day 0. A piece of gauze with a live *S. aureus* suspension (105–108 CFU) was attached to the wound to form an acute wound model. Due to the wireless, flexible, and lightweight design of the SWPS, in vivo evaluations were performed in freely moving animals wearing our devices (Figure 5b; Movie S2, Supporting Information). No significant difference in movement tracing was observed in the SWPS‐attached animals compared to the controls, indicating a comfortable therapeutic modality for future clinical use (Figure 5c). In this in vivo test, our device could easily detect different *S. aureus* infections at varying concentrations, further confirming the high sensitivity of the SWPS (Figure S12, Supporting Information). Using our system, increasing *S. aureus* infections were recorded in the first 4 days after wound creation, as shown in Figure 5d,e. We then applied electrical stimulation to evaluate the wound‐healing function of the SWPS device. Substantially higher rates of wound closure were observed in the electrically treated wounds than in the untreated control group (Figure 5d,f). Due to the wireless low‐power design of our device, the infection status could be queried on‐demand, and different electrical stimulation protocols could be easily adjusted in our system via a mobile phone, which could be further used in personalized therapy (Figure S13 and Movie S3, Supporting Information). For example, different current intensities or stimulation durations could be readily delivered wirelessly using the proposed system. As shown in Figure 5f and Figure S14 (Supporting Information), electrical stimulation at 100 and 300 µA with 30 min duration was identified to be the most effective protocol for the acute wound rat models used in this study. Moreover, the reactive tissue response to our SWPS device was assessed using immunohistochemical analysis of the sections of full‐thickness skin wounds via Masson's trichrome (MT) staining and Hematoxylin & Eosin (H&E) staining (Figure 5g–i). In the one‐week assessment, significantly higher collagen deposition and granulation tissue formation were observed in the electrical stimulation (ES) groups, as shown by MT staining (Figure 5g). Notably, a lower distribution of inflammatory cells within the wound bed was further quantified in the ES groups compared to the control groups, indicating that continuous electrical pulses delivered via our device significantly improved functional tensile recovery (Figure 5h,i). Overall, these results suggested successful closed‐loop management of wounds in rats, further exemplifying the power of the proposed wireless intelligent patch for monitoring wound conditions and delivering timely treatment.

## 3 Discussion

In this study, we have developed a SWPS capable of wireless, closed‐loop, in‐situ management of chronic wounds. This integrated wearable system combined a microfluidic structure, OECT‐based biosensors, an ES strategy, and flexible electronic technology to enable in‐situ wound infection monitoring and timely treatment delivery. Real‐time monitoring of bacterial virulence in both in vitro and in vivo settings was successfully achieved using the SWPS. Furthermore, the feasibility of SWPS technology for accelerating wound healing in vivo was demonstrated by coupling it with programmed electrical cues.

Flexible electronic systems have recently emerged as powerful tools for detecting various biomarkers in human sweat, interstitial fluid, and wound exudate.^[^ ^12^, ^19^ ^]^ To realize real‐time monitoring of the wound conditions, most previously demonstrated sensors focused on monitoring small molecules, ions, or electronic transductions such as impedance, temperature, humidity, and pH.^[^ ^10^, ^20^ ^]^ However, direct monitoring of bacterial virulence at the wound site remained challenging due to the complexity of readout instrumentation and module integration.^[^ ^21^ ^]^ To overcome this issue, we employed a DNAgel‐based OECT sensor for in‐situ monitoring of bacterial virulence.^[^ ^22^ ^]^ Specifically, unlike traditional OECT readouts that rely on faradaic redox properties, our device detected changes in *I*_ds_ induced by the DNAgel‐coated gate when exposed to extracellular DNase secreted by opportunistic pathogens at the wound sites. While DNA hydrogel‐based patches have been demonstrated to detect *S. aureus*, our device showed a lower detection limit (104 CFU in *S. aureus* suspension) due to the OECT assembly.^[^ ^23^ ^]^ Moreover, compared to physical cross‐linking, which required cold‐hot cycling conditions, the cross‐linking method introduced in this study did not involve such experimental conditions and could provide a more stable hydrogel network through covalent bonds.^[^ ^24^ ^]^ As shown in Figure S9 (Supporting Information), our devices could be stored at room temperature with 90% humidity for eight days while maintaining good detection performance. Therefore, the fabrication and storage of our OECT device were relatively cost‐effective and straightforward. This strategy avoids the need for complicated readout instrumentation, enables low‐cost system design and fabrication, and significantly improves sensor sensitivity.^[^ ^25^ ^]^ It also paved the way for in‐situ detection of other microbial markers using advanced programmable hydrogel.^[^ ^26^ ^]^

While significant progress has been made in wound monitoring and treatment platforms, most existing methods focus solely on either detection or treatment.^[^ ^8^, ^20^, ^27^ ^]^ Additionally, current wound management systems face challenges related to the controllability and visualization of wound infection. To address these issues, we integrated an electrical circuit design with a mobile phone app featuring intuitive human‐computer interaction in the SWPS. This combination allowed for real‐time monitoring of infection and enabled programmable electrical therapy tailored to the patient's individual needs. The flexible design, miniaturized scale, long communication range, low power consumption, and high data collection accuracy of our system made it well‐suited for use in various complex environments.

As a proof‐of‐concept study, we have demonstrated the functionality of the SWPS technique for the closed‐loop management of wounds in rodents. It is noteworthy that this system holds potential for translation to various other bio‐applications, such as glucose management,^[^ ^28^ ^]^ regulation of intraocular pressure,^[^ ^29^ ^]^ and treatments for oral diseases.^[^ ^30^ ^]^ Indeed, several areas for further improvement exist. For example, integrating more advanced microfluidic structures or microneedle arrays for programmable drug delivery would significantly enhance biochemical treatments in animals.^[^ ^31^ ^]^ Additionally, multiple further in‐depth studies, including biocompatibility tests, long‐term evaluation, and efficacy assessment in non‐rodent animals and primates, are warranted. With these promising future directions in mind, the results of our study establish a highly effective, fully programmable, and easily implementable platform for personalized monitoring and treatment of chronic wounds.

## 4 Experimental Section

### Fabrication of FPCB

One of the key components of our system is a FPCB (Figure S2a,b, Supporting Information). A flexible copper‐clad laminate (HAREKO50513D; Hengchi) was utilized as the substrate. The circuit was defined on copper using laser ablation. Subsequently, a cover layer (HCF1025; Hengchi) was bonded to the circuit board, resulting in an FPCB. The exposed part of the electrode surface was used for Au deposition with a thickness of 1 µm, creating a prototype electrode. Lead‐free low‐temperature soldering paste (LF999; KELLY SHUN) was used to bond various surface‐mounted components, including the BLE SoC (nRF52832; Nordic Semiconductor), amplifier (TL084; Texas Instruments), 0402‐sized surface‐mounted capacitors and resistors, power management chip (TP7660; TOPPOWER), a constant current source (LM334DT; STMicroelectronics), and 2.4 G surface‐mounted ceramic antenna (CA‐C03; CrossAir) by heating at 230 °C (Figure S2c, Supporting Information).

### Fabrication of OECT Module

The preliminary shape of the electrode was designed using Altium Designer and fabricated based on an FPCB using the method. A magnetron sputtering device was used to sputter a 20 nm thick Pt layer followed by a 200 nm thick Au layer onto the exposed pad of the FPCB, serving as the source, drain, and gate of the OECT (Figure S5, Supporting Information). For the PEDOT: PSS semiconducting layer, a solution consisting of 93.75% (v/v) PEDOT: PSS (Clevios PH1000; Heraeus Deutschland), 5% (v/v) ethylene glycol (EG) (E103323; Aladdin), 1% (v/v) 3‐glycidoxypropyltrimethoxysilane (GOPS) (G107576; Aladdin), and 0.25% (v/v) 4‐dodecylbenzenesulfonic acid (DBSA) (D106550; Macklin) was prepared. After mixing the components to form a suspension, the mixture underwent ultrasonic treatment for 10 min, followed by filtration using polyvinylidene difluoride (PVDF) filters with a pore size of 22 µm (brand: JINTENG). The filtrate was then stored at a stable temperature of 4 °C for further use. An ultrathin polyimide tape mask (5413; 3 m) with a specific pattern, prepared using a laser marking machine (MUV‐E‐S12; Ymlaser), was adhered between the source and drain electrodes. Subsequently, following air plasma treatment of these electrodes, the mask was removed, resulting in the formation of channel regions with different wettability patterns. Finally, the semiconducting layer was formed by depositing the PEDOT: PSS mixture onto the channel region after heating at 120 °C for 60 min.

### Synthesis of DNA Hydrogel

The DNA hydrogel was prepared using a physical‐chemical cross‐linking method. Ethylene glycol diglycidyl ether (EGDE) served as the cross‐linking agent, reacting with the amino groups in the nucleobases of deoxyribonucleic acid sodium salt from salmon testes (smDNA). This chemical cross‐linking process was catalyzed by N,N,N“,N”‐Tetramethylethylenediamine (TEMDE), resulting in a DNA hydrogel with a 3D network structure. Different concentrations of smDNA (4, 6, 8, 10, and 12 wt.% (D1626; Sigma)) were individually added to 4 mm NaBr (S112315; Aladdin). After thorough mixing, 10% EGDE (E823392, Macklin) by weight of smDNA and 0.55 wt.% TEMED (N818999, Macklin) were added to the precursor solutions. These components were uniformly mixed with the precursor solution and transferred into a plastic syringe. The mixture was allowed to cross‐link at 4 °C for 1 h to obtain the hydrogel precursor. Subsequently, the hydrogel precursor was extruded onto the desired surface of the device. Following this, a 4‐h heating process at 50 °C with 90% humidity was conducted. The final DNA hydrogel was obtained by cooling the device to room temperature.

### Fabrication of Microfluidic Layer

A microfluidic structure was designed and integrated into the SWPS for wound exudate collection. This structure comprised three thin polyethylene terephthalate (PET) layers stacked and adhered together. It consisted of a bottom layer designed for wound exudate collection, a middle layer with microfluidic channels, and an upper gate channel layer. All three layers were fabricated using laser cutting techniques and assembled using double‐sided PET tape, resulting in a flexible microfluidic structure.

### Integration of SWPS

The 8 mm diameter silver column was secured at the designated position on the sensor through reflow soldering. Subsequently, the microfluidic structure was affixed to the modified OECT sensor. The DNA hydrogel precursor was then applied to the gate of the OECT and subjected to heating at 50 °C with 90% humidity for 4 h. Following the cooling process, the final SWPS was obtained and ready for further testing.

### Morphological Characterization of DNA Hydrogel

The morphological stability of the DNA hydrogels was assessed through microimaging on a polyimide (PI) substrate. The DNA hydrogel was immersed in PBS for 80 min at room temperature. A stereomicroscope (NSZ‐606; Jiang Nan) was used to capture images of the DNA hydrogel and assess its swelling behavior. For the DNA hydrogel dissolution test, a DNase I solution was prepared with a concentration of 0.1 U mL^−1^ by combining 10% (v/v) DNase I (EN0521; Thermofisher) storage solution and 10% (v/v) 10× reaction buffer (25 mm MgCl_2_, 1 mm CaCl_2_) with PBS solution. Subsequently, the DNA hydrogel was exposed to the DNase I solution in a 37 °C water bath to facilitate the digestion process. The morphologies of the DNA hydrogels were then evaluated using a stereomicroscope. ImageJ software was used to analyze the morphological changes of the DNA hydrogel. The volume of the hydrogel was estimated using the formula for a sphere segment.

### EIS Analysis of the DNA Hydrogel

Electrochemical impedance spectroscopy (EIS) was conducted using an electrochemical workstation (CHI660E, Chinstruments) in a three‐electrode system. An Ag/AgCl (CHI111, Chinstruments) was used as the reference electrode, while a platinum plate electrode (5 × 5 × 0.2 mm, GaossUnion) was used as the counter electrode. The working electrode consisted of a GCE (3 mm diameter, GaossUnion). Prior to measurements, the electrodes were polished and immersed in a solution mixture of 1 mm K3Fe(CN)6/K4Fe(CN)6 (1:1) and 0.1 mm KCl post‐polishing. EIS measurements were conducted over a frequency range of 0.1–100 kHz, with a DC potential of 0.2 V and an amplitude of 10 mV. First, a bare GCE was immersed in the solution for EIS scanning. Second, the DNA hydrogel was applied to the surface of the GCE, and EIS scanning was performed once the hydrogel reached a stable state of swelling. The GCE coated with the DNA hydrogel was then exposed to a 0.1 U mL^−1^ DNase I solution to simulate DNA enzyme digestion for durations of 30 and 60 min, followed by additional EIS scans. The obtained EIS results were subsequently fitted using the Randles equivalent circuit model in the ZView software. In this model, *R*_ct_ represents the charge transfer resistance, which characterizes the rate of charge transfer in the electrolyte, while CPE signifies the non‐ideal capacitive behavior formed between the electrode surface and the electrolyte (Figure 3f).

### In Vitro Test with DNase I Solution

DNase I solutions of varying concentrations were prepared by diluting the DNase I storage solution (1 U mL^−1^) into 0.01 m PBS containing 2.5 mm MgCl_2_ and 0.1 mm CaCl_2_ at dilution ratios of 10%, 5%, 2.5%, 1%, and 0.05%. Concurrently, a control group consisting of a PBS solution containing all components except DNase I was prepared. Six groups (five devices in each group), were immersed in PBS solution for 1 h, during which OECT characterization measurements were conducted at 20‐min intervals to establish the baseline. Subsequently, the prepared DNase I solution was added to the devices. The readouts of the OECT characterizations were recorded using a digital source meter (2612B, Keithley). The measurement parameters were set as follows: i) the drain‐source current (*I*_ds_) was collected over a 50‐s period with the gate voltage (*V*_g_) set to 0.1 V and the drain‐source voltage (*V*_ds_) at 0.1 V; ii) the transfer curves of the OECT were recorded with *V*_g_ ranging from 0.1 to 0.75 V while keeping *V*_ds_ at 0.1 V. The same parameters and methods were applied for subsequent OECT characteristic analysis.

### In Vitro Sensing of Bacteria

Two types of bacterial strains were utilized in this study: i) DNase‐positive strains, comprising SA (ATCC 25923), and ii) DNase‐negative control strains, including SE (CMCC 26069), EC (ATCC 25922), PA (ATCC 27853), and CA (ATCC 6919). Suspensions of these bacterial strains in PBS were prepared at a concentration of 107 CFU mL^−1^ using a spectrophotometer. To inoculate the bacteria onto the DNase test agar, the agar streak plate method was used. The plates were incubated at 37 °C for 24 h, followed by covering 1 m hydrochloric acid on the surface. If the plates were presented for clear zones around the colonies, it indicated that the bacterial strain is DNase‐positive (Figure S15, Supporting Information).

For the OECT tests, the devices were initially prepared and calibrated in PBS. Subsequently, the devices were transferred to suspensions of *S. aureus* (103, 104, and 105, 106 CFU mL^−1^), as well as suspensions of DNase‐negative control strains (106 CFU mL^−1^). Changes in drain‐source current (*I*_ds_) were then recorded for analysis.

### In Vitro Test of Bacterial Responses of Electrical Stimulation

Hundred microliters of *S. aureus* at a concentration of 107 CFU mL^−1^ was inoculated onto LB agar plates. Subsequently, pure silver electrodes (99.99%) (≈8 mm in diameter) were inserted into the agar plates. A 300 µA current was applied through the electrodes for 30 min. The control group underwent silver electrode insertion without ES. Following incubation at 37 °C for 24 h, the bacterial growth near the silver electrodes was analyzed. Moreover, the samples of agar around the electrodes were extracted using a biopsy punch with a diameter of ≈4 mm. These samples were then placed in tryptic soy broth (TSB) and incubated at 37 °C on a shaking incubator for 3 h to obtain a suspension. The absorbance of the suspensions at different wavelengths was measured using a spectrophotometer (Figure S16, Supporting Information).

### In Vivo Tests of SWPS

Male Sprague–Dawley rats (7–8 weeks, 250–280 g in weight) were used in this study and were housed in a room with a 12‐h/12‐h light/dark cycle, with ad libitum access to food and water. All animal procedures were conducted following the guidelines approved by the Institutional Animal Care and Use Committee of Sun Yat‐sen University (Ref: SYSU‐IACUC‐2019‐B1004).

The dorsal skin on the back of the rat was prepared using animal razors and depilatory fluid (PH1877; Phygene). Full‐thickness wounds (≈8 mm in diameter) were created on the dorsal skin using a biopsy punch (BP80; EDM3 SOLUTIONS).

For the in vivo wound infection sensing test, the wounds were inoculated with 50 µL of *S. aureus* suspensions at different concentrations (total counts of 105, 106, 107, and 108 CFU). Concurrently, 50 µL of sterile PBS was inoculated into the wounds as a blank control. The wounds were immediately covered with Tegaderm Film (3 m) post‐inoculation. Upon confirmation of the successful colonization of *S. aureus* on the wounds, the SWPS was applied to record the OECT readouts.

For the in vivo wound‐healing test: 106 CFU of *S. aureus* was inoculated onto the wounds of rats. ES experiments were conducted daily, with the control group receiving no additional treatment, and their Tegaderm Film dressings being changed regularly. A 30 min of 300 µA ES treatment was administered to the animals daily, with the anode placed on the wound and the cathode on the adjacent normal skin. The current was supplied by the device, and parameters were adjusted using a mobile phone. Post‐treatment, the wounds were covered with Tegaderm Film and daily photographs were captured to document wound appearance.

### Tissue Processing and Imaging

On the 5th day of the in vivo experiment, wound tissue sections were meticulously prepared and subjected to staining with HE as well as Masson's trichrome. Initially, the wound site and surrounding skin were meticulously isolated and fixed in a 4% polyformaldehyde solution. Subsequently, the samples were embedded in paraffin (39 601 006; Leica), sectioned using a Leica HistoCore MULTICUT instrument, and stained with (H&E) and MT. Tissue slices were then washed three times with PBS, with each wash cycle lasting 10 min at room temperature. The stained tissue slices were carefully mounted onto glass slides for imaging purposes. Imaging was conducted utilizing a fully automated inverted fluorescence microscope (Leica THUNDER DMi8), which was equipped with a cooled high‐speed sCMOS camera (DFC 9000GT) and a 10× (NA, 0.4) objective. The LAS X software served as the control interface for the microscope during imaging sessions.

### Software/App Development

The software development process encompassed two primary parts: i) The embedded system tailored for the nRF52832 SoC and ii) the mobile phone application crafted for user interaction. The firmware for the nRF52832 was written in C and utilized the Nordic SDK. This framework facilitated the efficient operation of the device in low‐power mode. Specifically, the firmware enabled the device to receive commands from a supervisory computer system, administer specified current outputs for ES, generate an excitation voltage for the OECT, and relay collected current signals back to the supervisory system.

On the mobile front, an application was developed using Android Studio and coded in Java. This application provides Bluetooth connectivity, enabling it to identify and connect with the devices. It grants users the ability to dispatch testing and ES commands, visualize real‐time data, perform data analysis, and receive alerts or warnings based on predefined thresholds.

### Statistical Analysis

Statistical analyses were performed using SPSS Statistics 25 (IBM Corp., Armonk, NY) or Origin 2021 (OriginLab Corp., Northampton, MA). The bar and curve graph data were presented as the mean ± standard deviation (SD). For the box plots, the vertical centerline indicated the median, while the width of the box and error bar represented the interquartile range (IQR) and 1.5 times the IQR, respectively. A two‐tailed paired student *t*‐test or an independent samples *t*‐test was used to calculate differences between the two groups. Differences were considered statistically significant at ^*^ *p* < 0.05, ^**^ *p* < 0.01, and ^***^ *p* < 0.001. Unless otherwise specified, the data shown are means ± SD. No blinding was used.

## Conflict of Interest

The authors declare no conflict of interest.

## Author Contributions

Z.L. and H.S. contributed equally to this work. X.L. conceived and coordinated the project. Z.L. and H.S. designed and fabricated the SWPS, and performed the in vitro and in vivo tests. Y.Z. and D.D. assisted in the analysis of the video and photograph recordings of animals. G.L. A.Y., Y.L., J.D., Y.Z., and K.J. contributed to data analysis and helped with writing. W.Z., Y.F., J.Y., and W.Z. contributed to bacteria‐related tests. Z.L., H.S., G.L., and X.L. wrote the manuscript.

## Supporting information

Supporting Information

Supplemental Movie 1

Supplemental Movie 2

Supplemental Movie 3

## Data Availability

The data that support the findings of this study are available from the corresponding author upon reasonable request.
