# Supplementary material for: Wireless Intelligent Patch for Closed‐loop In Situ Wound Management
Source: Adv Sci (Weinh). 2024 Jun 3;11(29):2400451. doi: 10.1002/advs.202400451 (PMC11304288; doi:10.1002/advs.202400451)
Supplement: Supplementary file 1 — Supporting Information [file ADVS-11-2400451-s004.pdf]

ADVANCED SCIENCE
Open Access

Supporting Information

for *Adv. Sci.*, DOI 10.1002/advs.202400451

Wireless Intelligent Patch for Closed-loop In Situ Wound Management

*Zijian Liu, Hao Song, Guanming Lin, Weicong Zhong, Yang Zhang, Anqi Yang, Yuxin Liu, Junhan Duan, Yajing Zhou, Kangjian Jiao, Donghai Ding, Yanwen Feng, Jun Yue, Wenjing Zhao and Xudong Lin**

Wireless Intelligent Patch for Closed-loop In-Situ Wound Management

Zijian Liu[1,3], Hao Song[1,3], Guanming Lin[1], Weichong Zhong[2], Yang Zhang[1], Anqi Yang[1], Yuxin Liu[1], Junhan Duan[1], Yajing Zhou[1], Kangjian Jiao[1], Donghai Ding[1], Yanwen Feng[1], Jun Yue[1], Wenjing Zhao[2], Xudong Lin[1*]

[1]Guangdong Provincial Key Laboratory of Sensor Technology and Biomedical Instrument
School of Biomedical Engineering
Shenzhen Campus of Sun Yat-Sen University
Shenzhen, 518000, China

[2]Shenzhen Key Laboratory for Systems Medicine in Infammatory Diseases,
School of Medicine
Shenzhen Campus of Sun Yat-Sen University
Shenzhen, 518000, China

[3]These authors contributed equally to this work

*Correspondence should be addressed to

Dr. Xudong Lin, linxd37@mail.sysu.edu.cn

Supplementary Figures

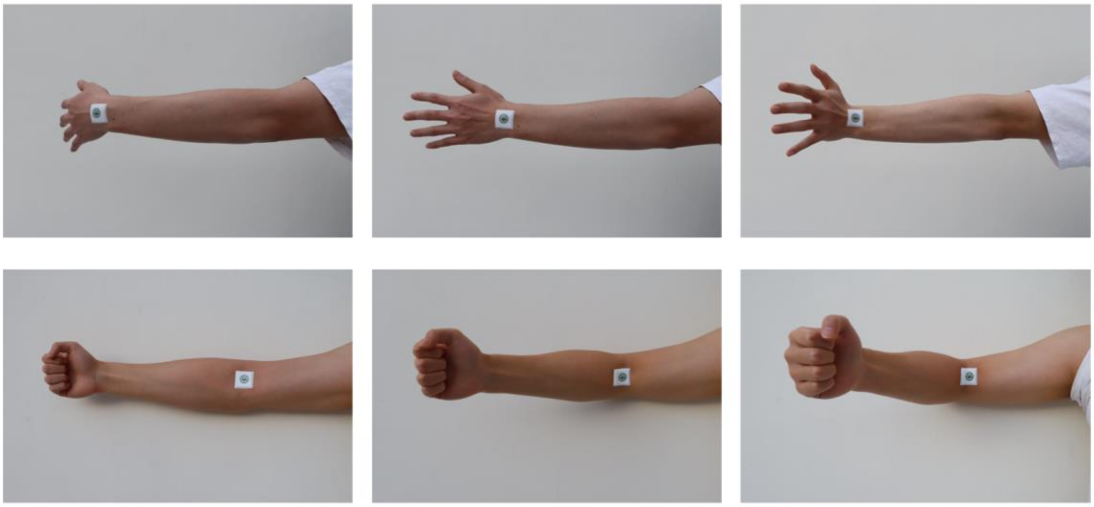

Supplementary Figure S1. Photographs showing the SWPS adhered to the wrist and the elbow.

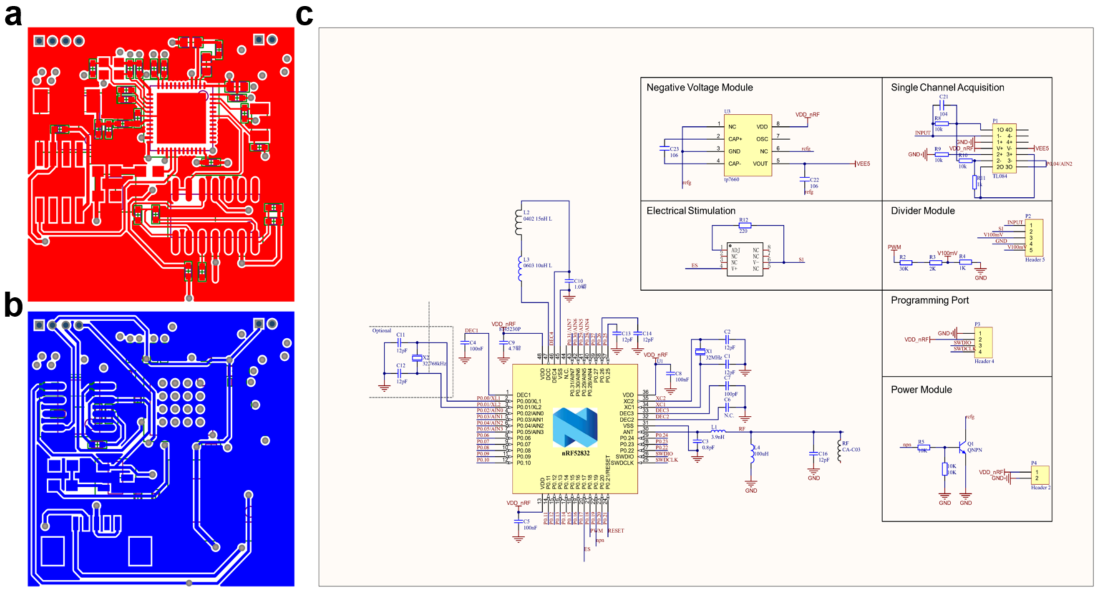

Supplementary Figure S2. **The FPCB design**. **a-b**, Top (**a**) and bottom (**b**) FPCB designs containing component layout and wiring conditions. **c**, Schematic diagram showing the nRF52832 based electrical circuits in our design.

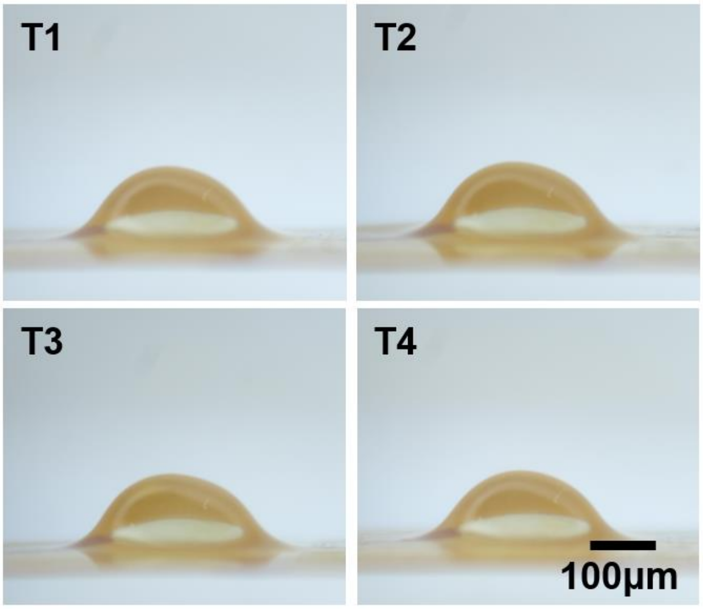

Supplementary Figure S3. **Stability of the DNA hydrogel.** Photographs showing the shape of DNA hydrogel in 1 × PBS environment. Imaging interval was 20 minutes.

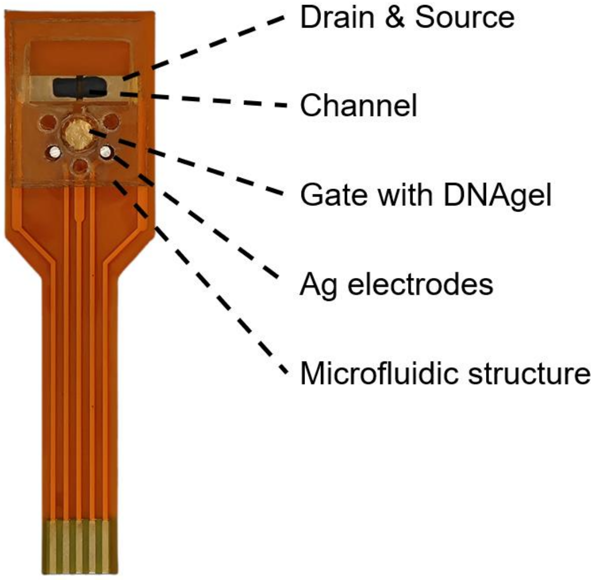

Supplementary Figure S4. Design of the integrated device. Photograph showing components of the FPCB based sensor, including an OECT with drain, source, semiconducting channel and gate, Ag electrodes and microfluidic structure.

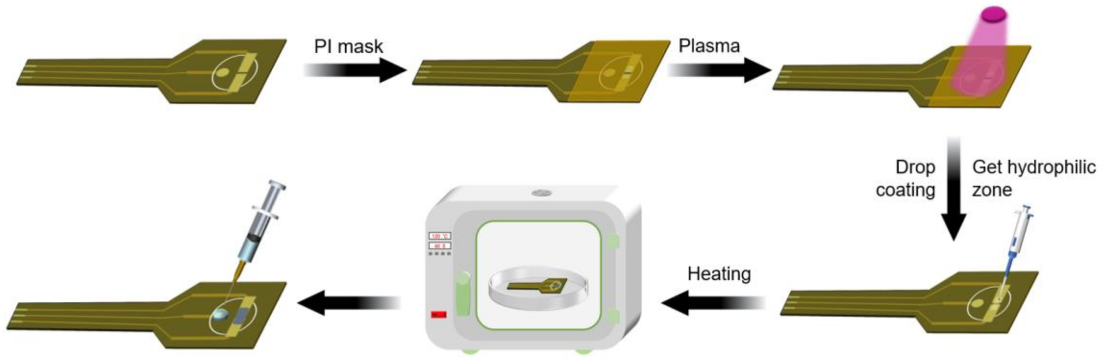

Supplementary Figure S5. **Schematics showing the fabrication of the OECT module.**

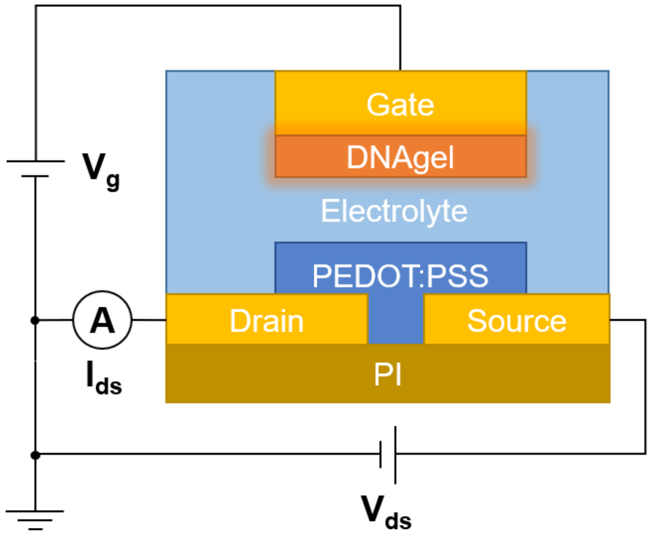

Supplementary Figure S6. **Schematic showing the OECT structure.** The OECT consisted of drain, source, gate, channel, electrolyte and the DNAgel. V_g (gate voltage) and V_{ds} (drain-source voltage) could be applied to regulate the channel current I_{ds}, which was used to evaluate the wound infection.

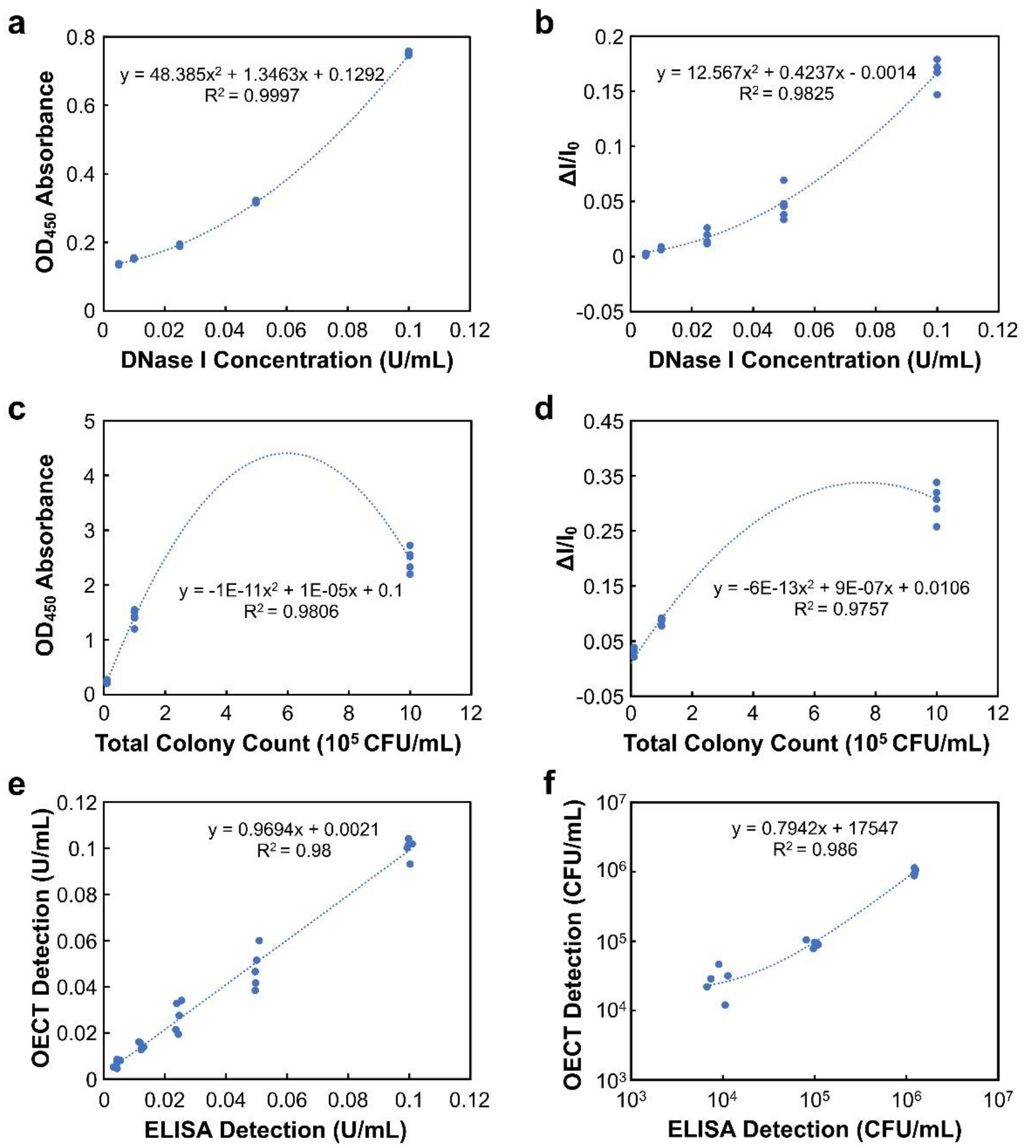

Supplementary Figure S7. **Comparison of the DNase I detection between the OECT based strategy and ELISA based method. a-b**, Detection results for DNase I by ELISA kit (**a**) and OECT system (**b**). **c-d**, Detection results for Staphylococcus aureus suspension by ELISA kit (**c**) and OECT system (**d**). **e-f,** Scatter plots comparing detection results, with ELISA measurements and OECT measurements for DNase I (**e**) and Staphylococcus aureus suspensions (**f**).

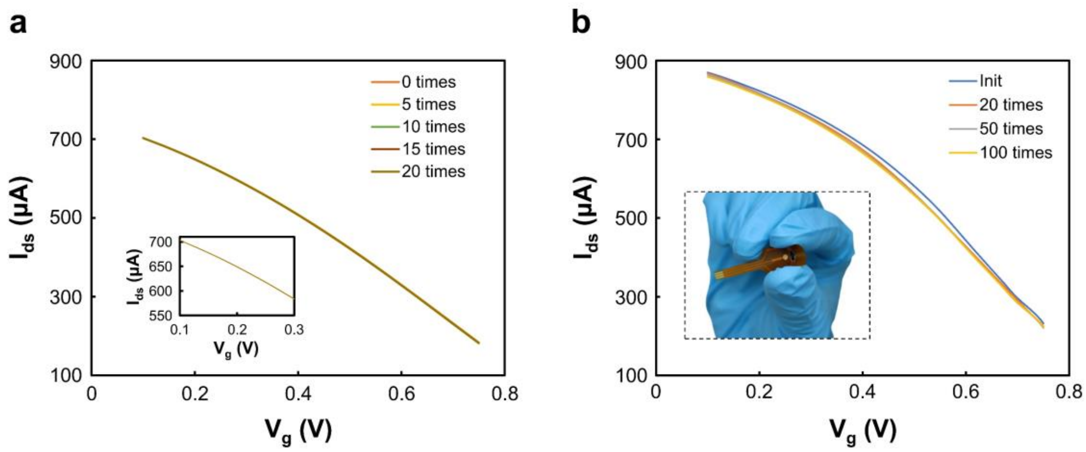

Supplementary Figure S8. **Stability tests of the sensing module. a**, The transfer curve of the sensor in repeated scans. **b**, The transfer curve of the sensor with repeated longitudinal bending.

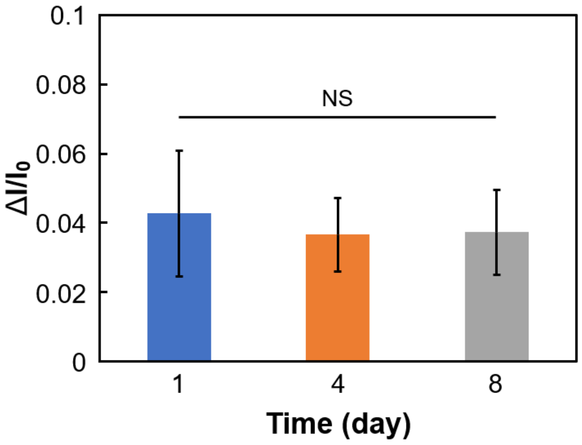

Supplementary Figure S9. **Long-term functional evaluation of the OECT module**. Normalized transient deviation of the I_{ds} was recorded in a DNase environment at the concentration of 0.05 U/mL *in vitro*. Two-tailed paired student t-test, n = 5, collected from 5 devices; NS, not significant; error bars indicated the Standard Deviation.

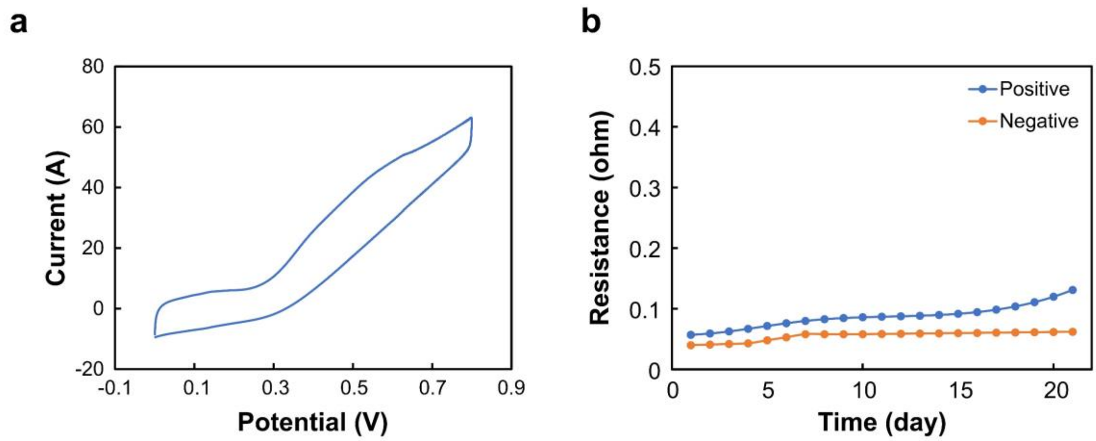

Supplementary Figure S10. **Electrical performance of the Ag electrodes in the SWPS**. **a**, Cyclic voltammogram of a pair of Ag electrodes as an electrical stimulator during immersion in DPBS (pH 7.4) at room temperature. **b**, Long-term evaluation of the resistances of Ag electrodes for electrical stimulation (positive and negative) with an applied voltage of 300 mV for 30 min/day during immersion in DPBS (pH 7.4) at 37 °C.

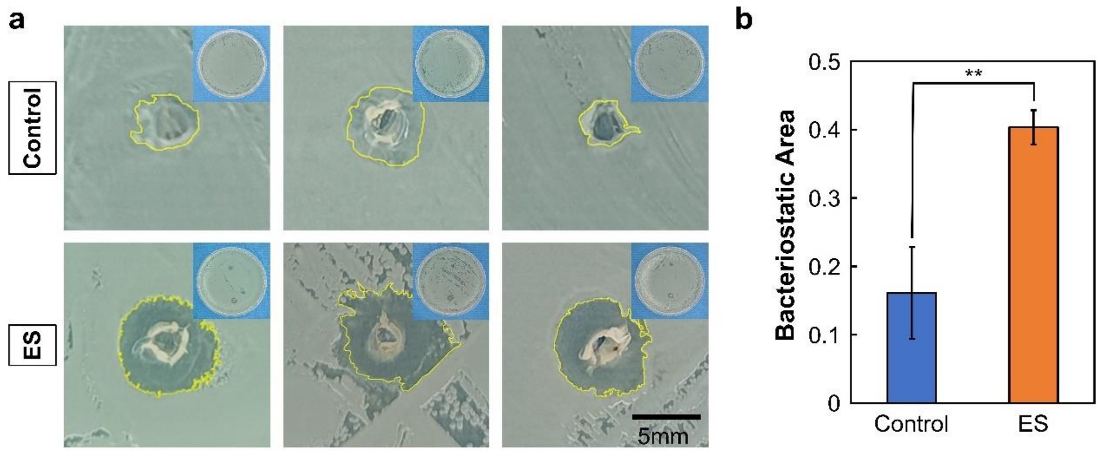

Supplementary Figure S11. **Electrical stimulation induced inhibition of bacteria growth *in vitro* using Ag electrodes. a**, Images showing the bacteria growth around the Ag electrodes without electrical stimulation (Control) and with a 300 μA DC current electrical stimulation (ES) for 30 minutes. **b**, Statistical results of the bacteriostatic area around the electrodes. Two-tailed paired student t-test, n = 3, collected from 6 plates; **$p < 0.01$; error bars indicated the Standard Deviation.

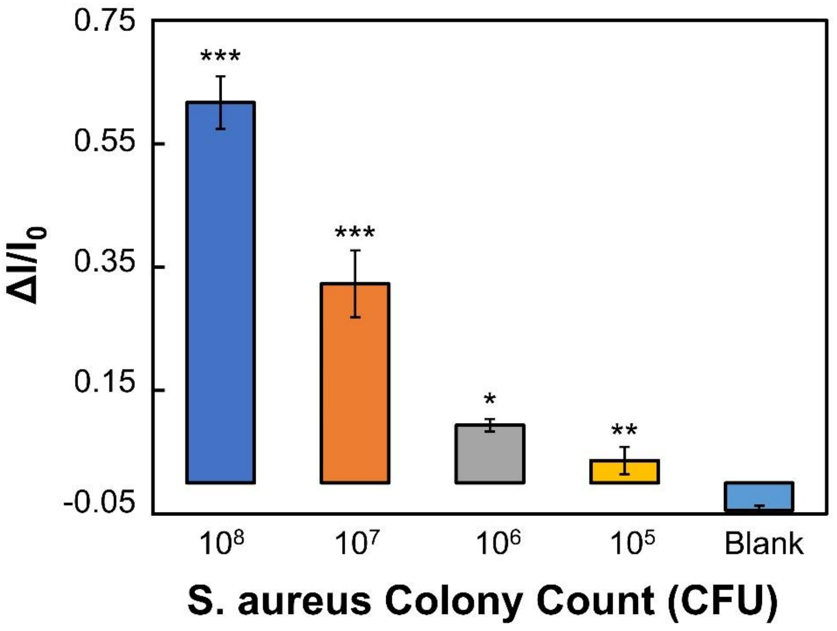

Supplementary Figure S12. ***In vivo* evaluation of the sensitivity of the SWPS.** I_{ds} deviation observed following the inoculation of wounds with varying concentrations of Staphylococcus aureus suspensions *in vivo*. The results were recorded one day post-inoculation. Two-tailed paired student t-test, n = 5, collected from 25 wounds; *$p < 0.05$, **$p < 0.01$, ***$p < 0.001$; error bars indicated the Standard Deviation.

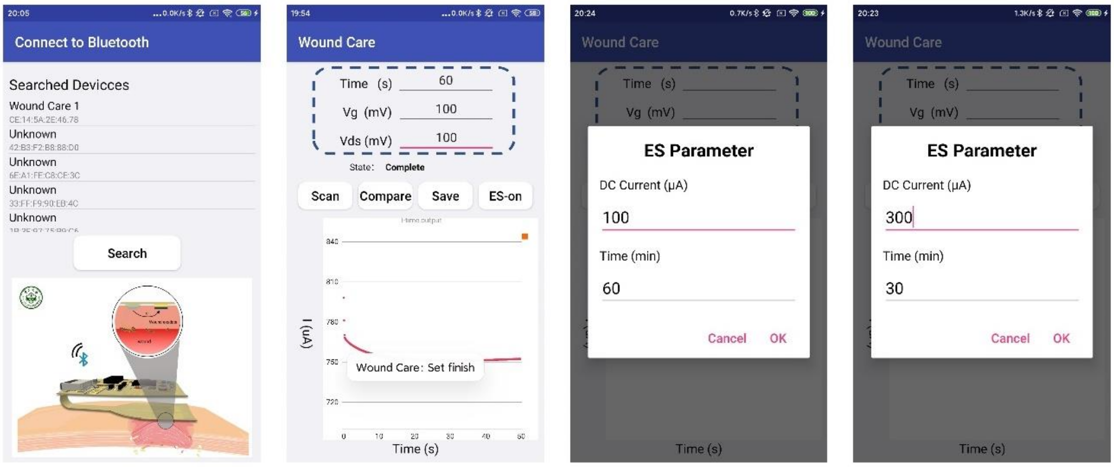

Supplementary Figure S13. **Screenshots of the mobile App interface**. Screenshots presented the adjustable settings for detection parameters and electrical stimulation parameters in the SWPS device. Users could easily connect to Bluetooth, detect signals from sensor and adjust electrical stimulation settings in the mobile phone App.

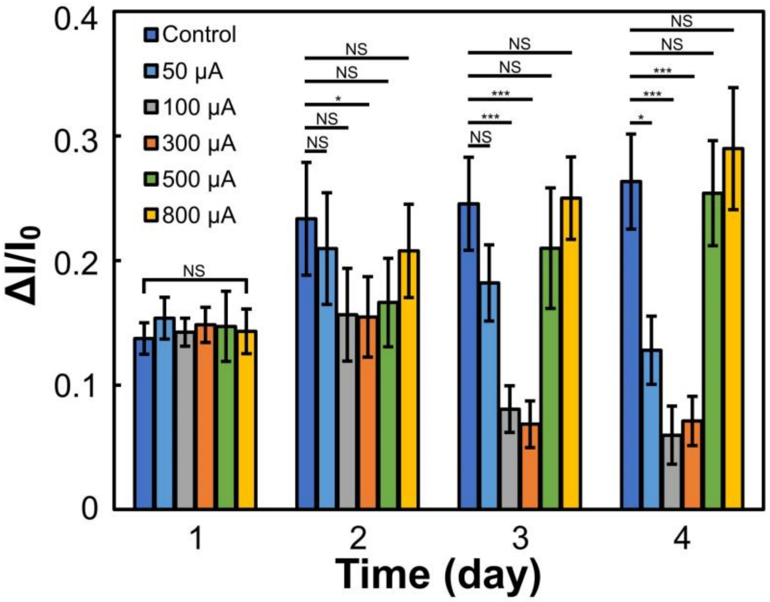

Supplementary Figure S14. **Impact of electrical stimulations with different current intensities on wound healing *in vivo*.** Stimulation duration, 30 minutes/day. Two-tailed paired student t-test, n = 5, collected from 5 devices; NS, not significant, *p < 0.05, **p < 0.01, ***p < 0.001; error bars indicated the Standard Deviation.

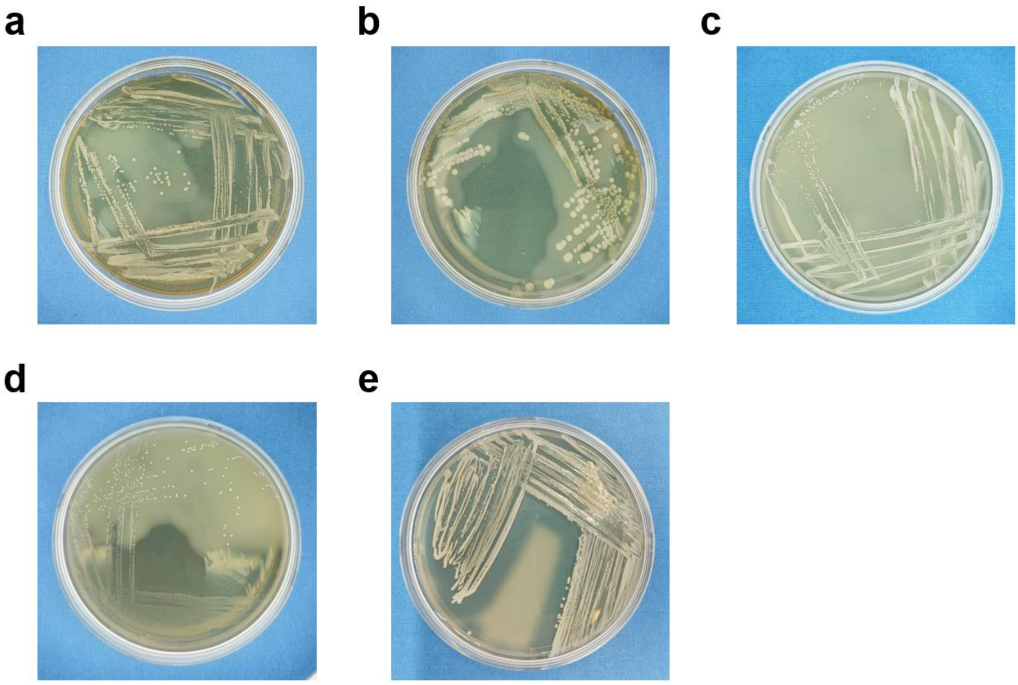

Supplementary Figure S15. **DNase responsive tests of different bacteria on deoxyribonuclease test agar. a-e**, The test was performed using 5 different bacteria including Staphylococcus epidermidis (**a**), Escherichia coli (**b**), Cutibacterium acnes (**c**), Pseudomonas aeruginosa (**d**), Staphylococcus aureus (**e**).

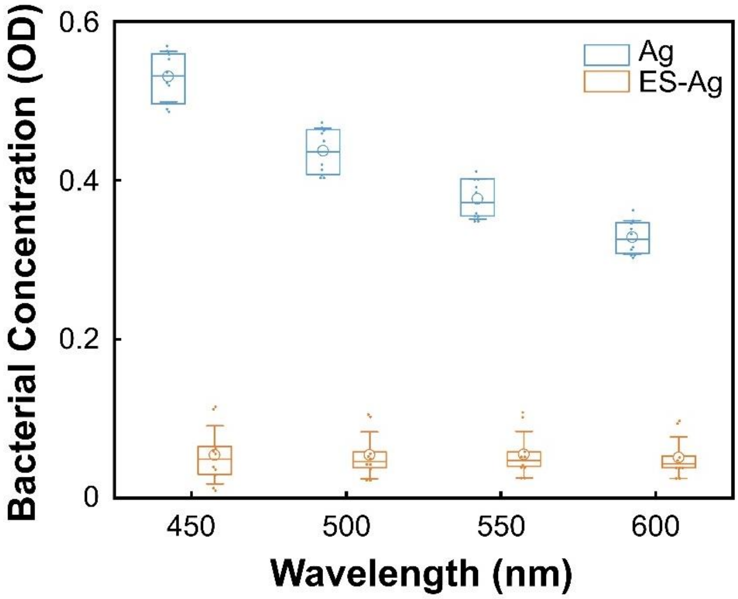

Supplementary Figure S16. **Evaluation of Staphylococcus aureus growth on LB agar plates with silver electrodes.** Electrodes were either electrically stimulated or unstimulated as controls.

Supplementary Movies

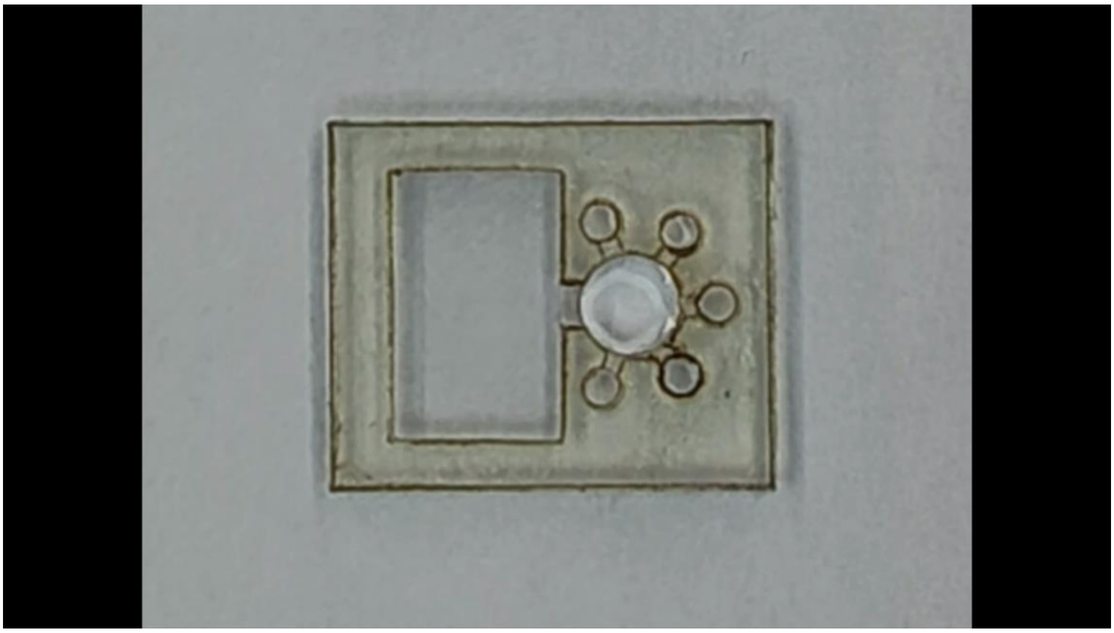

Supplementary Movie S1. Microfluidic layers for wound exudate (blue dye) collection.

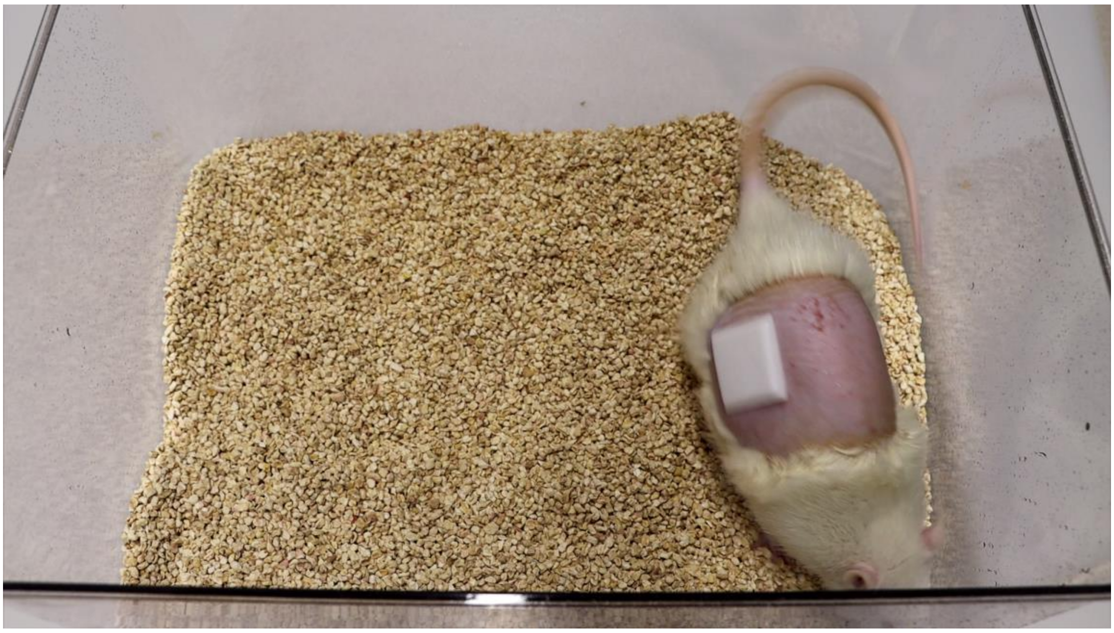

Supplementary Movie S2. A freely moving rat with the SWPS.

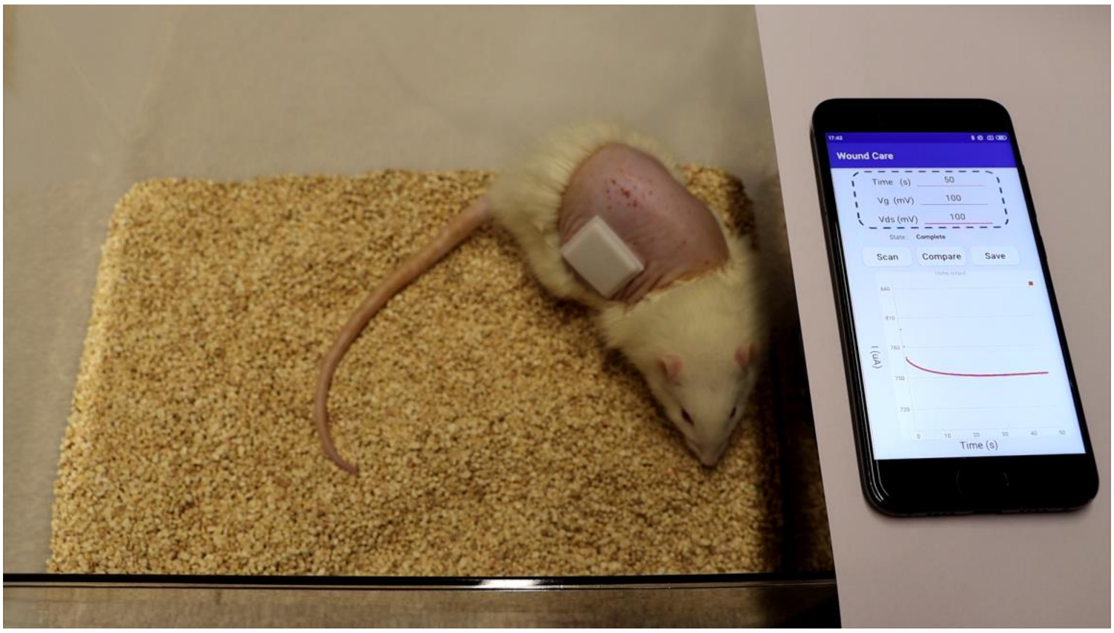

Supplementary Movie S3. ***In vivo*** **wound monitoring using the SWPS.**
